# Influence of Intensity RAMP Incremental Test on Peak Power, Post-Exercise Blood Lactate, and Heart Rate Recovery in Males: Cross-Over Study

**DOI:** 10.3390/ijerph16203934

**Published:** 2019-10-16

**Authors:** Kamil Michalik, Kuba Korta, Natalia Danek, Marcin Smolarek, Marek Zatoń

**Affiliations:** Department of Physiology and Biochemistry, Faculty of Physical Education, University School of Physical Education in Wroclaw, 35 Paderewskiego Street, 51-612 Wroclaw, Poland; kuba.korta.kolarstwo@gmail.com (K.K.); natalia_danek@interia.pl (N.D.); marcin.cluby@interia.eu (M.S.); marek.zaton@awf.wroc.pl (M.Z.)

**Keywords:** incremental test, RAMP protocol, peak power, blood lactate, heart rate recovery

## Abstract

*Background:* The linearly increased loading (RAMP) incremental test is a method commonly used to evaluate physical performance in a laboratory, but the best-designed protocol remains unknown. The aim of this study was to compare the selected variables used in training control resulting from the two different intensities of RAMP incremental tests. *Methods:* Twenty healthy and physically active men took part in this experiment. The tests included two visits to a laboratory, during which anthropometric measurements, incremental test on a cycle ergometer, and examinations of heart rate and blood lactate concentration were made. The cross-over study design method was used. The subjects underwent a randomly selected RAMP test with incremental load: 0.278 W·s^−1^ or 0.556 W·s^−1^. They performed the second test a week later. *Results:* Peak power output was significantly higher by 51.69 W (*p* < 0.001; t = 13.10; ES = 1.13) in the 0.556 W·s^−1^ group. Total work done was significantly higher in the 0.278 W·s^−1^ group by 71.93 kJ (*p* < 0.001; t = 12.55; ES = 1.57). Maximal heart rate was significantly higher in the 0.278 W·s^−1^ group by 3.30 bpm (*p* < 0.01; t = 3.72; ES = 0.48). There were no statistically significant differences in heart rate recovery and peak blood lactate. *Conclusions:* We recommend use of the 0.556 W·s^−1^ RAMP protocol because it is of shorter duration compared with 0.278 W·s^−1^ and as such practically easier and of less effort for subjects.

## 1. Introduction

The incremental exercise test (with linearly increased loading) is a method commonly used to evaluate aerobic capacity in laboratory conditions [1]. It provides information on peak power output (PPO), maximal heart rate (HRmax), and peak blood lactate (La^−^), which are commonly used as criteria for attaining maximal exertion [2,3]. The incremental exercise test also allows the assessment of heart rate recovery (HRR) [4,5,6]. The monitoring of changes in the above-mentioned parameters is used in training control regardless of the sports level. However, the test protocol can influence exertion and physiological parameters [7]. Therefore, the conditions which provide reliable and valid measures of maximal and submaximal physiological variables are still sought.

Previous research compared the influence of time throughout the incremental testing stages. In their research, Machado et al. [8] obtained higher maximal speed and post-exercise La^−^ and lower HRmax in an 11-min test (1-min stages) in comparison to 19- and 26-min tests (2- and 3-min stages, respectively). Thus, it is necessary to search for a test protocol which does not last long and in which the level of fatigue will not grow too rapidly, also in the initial phase of the test. This solution is provided through the inductive braking system of a cycle ergometer. The inductive braking system of the cycle ergometer enables changes in the slope of the load increase curve, which allows for finding new ways of testing physical performance, e.g., linearly increased loading (RAMP) protocols [9,10,11]. Our previous work confirmed the validity of the protocol with RAMP compared to the traditional STEP protocol in young road cyclists. In RAMP, the load was increased by 0.278 W·s^−1^ (equivalent to 50 W·3 min^−1^) and obtained higher peak power, maximal oxygen uptake (VO_2_max), and lower post-exercise blood lactate concentration [9]. However, heart rate did not differ compared to STEP. Both tests were carried out for approx. 24 min. According to some authors, the incremental test duration should amount to 8 to 12 min (VO_2_max measurements in particular) due to thermoregulatory loading and dehydration in longer tests [12,13,14]. This may lead to fatigue and the premature termination of the test, all of which results in not attaining maximal values for the tested parameters [7].

In addition to the previously mentioned PPO, HRmax, and La^−^, examination of HRR can also be useful in monitoring response to training and programming training loads since there is no need of special equipment [15,16]. Post-exercise HRR drops to rest levels by reactivating the vagal nerve (parasympathetic nervous system). This may indicate functional disorders caused by fatigue, overtraining, or dehydration [15,16]. HRR is assessed by the absolute difference between the peak HR at the end of the exercise and HR after 60 s (HRR60) [17], which is a risk marker for cardiovascular mortality rate [18]. The impact of the load increase curve in RAMP on HRR in young, physically active males has not been yet investigated. Thus, it seems appropriate to verify if maximal exertion used in different linear protocols (RAMP) of incremental test changes physiological and biochemical responses, e.g., HRR.

Therefore, the purpose of this study was to compare the selected variables used in training control resulting from the two different intensities of RAMP incremental tests. The protocol was designed to induce fatigue after 8–12 min, thus a two-fold higher load increase was used compared to our previous study. It was hypothesized that the subjects would attain higher physiological and biochemical responses and quicker HR recovery in 0.556 W·s^−1^ protocol.

## 2. Material and Methods

### 2.1. Subjects

There were twenty (*n* = 20) healthy and physically active men who volunteered for the study. The participants provided their written, informed consent to participate. The subjects were physically active students without professional training history. Weekly physical activity in the last month before the experiment was collected through a questionnaire. Each subject was familiarized with the experiment protocol. Table 1 presents the selected anthropometric parameters and weekly physical activity of the examined competitors.

### 2.2. Study Design

The tests included two visits to the laboratory during which anthropometric measurements, incremental testing on the cycle ergometer, and examination of heart rate and blood lactate concentration were made. The cross-over study design method was used. The subjects underwent one randomly selected RAMP test with incremental load: 0.278 W·s^−1^ or 0.556 W·s^−1^. A week later they performed the second test. During this 7-day interval, subjects were asked to carry on their normal activities and not to change any exercise habits. For 24 h before testing sessions, participants were instructed to avoid strenuous physical activity. The experiment was carried out in the Stress Research Laboratory (PN-EN ISO 9001: 2001). The experiment was approved by the Research Ethics Committee (1/2019) and the study’s design adhered to the Declaration of Helsinki.

### 2.3. Incremental Testing

Prior to testing, both body mass and height were assessed using a WPT 200 medical scale (Radwag, Radom, Poland). Percent fat mass (%FM) was determined by near-infrared interactance using a 6100/XL analyzer (Futrex Tech, Inc., Gaithersburg, MD, USA) placed on the middle of the biceps brachii muscle of the dominant upper limb. The tests were performed on a stationary cycle ergometer Excalibur Sport (Lode BV, Groningen, The Netherlands).

**Figure 1:**
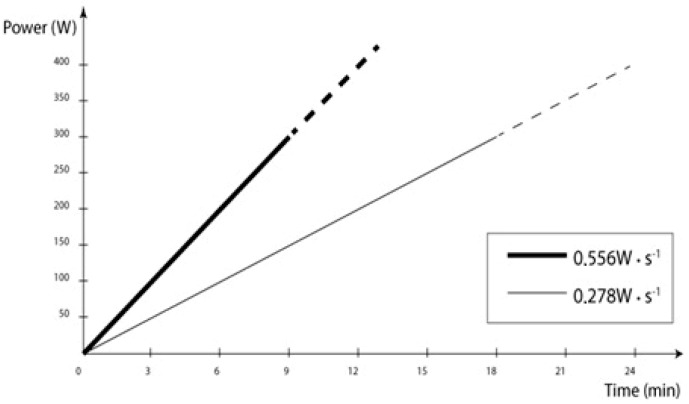
Linearly increased loading (RAMP) incremental testing protocols utilized in this study (thin line—0.278 W·s^−1^; bold line—0.556 W·s^−1^).

The following load increase protocols were used (Figure 1):

(1) Test 0.278 W·s^−1^—it started with 0 W load, which was increased linearly by ~0.278 W·s^−1^ which corresponds to 50 W·3 min^−1^.
(2) Test 0.556 W·s^−1^—it started with 0 W load, which was increased linearly by ~0.556 W·s^−1^ which corresponds to 100 W·3 min^−1^.

The minimum cadence was 60 rotations per minute (rpm). The test was continued until volitional exhaustion. Heart rate (bpm—beats per minute) was continually monitored by telemetry with an S810 heart rate monitor (Polar, Kempele, Finland) to determine resting values, maximal heart rate (HRmax), and heart rate recovery (HRR). The measurements were carried out two minutes prior test start and continued 5 min after termination. During rest, the subjects were in a sitting position. Arterialized capillary blood was drawn from the fingertip immediately before and 3 min after the test to determine peak blood lactate concentration (La^−^) (mmol·L^−1^) with photometric testing (Dr Lange 140 photometer: LP 400 Dr Lange, Berlin, Germany).

Maximal exertion was considered to be attained at the following criteria [2]: (1) La^−^ ≥ 8 (mmol·L^−1^); and (2) predicted HRmax ± 10 bpm from the formula (208–0.7∙age) introduced by Tanaka et al. [19] for healthy adults.

### 2.4. Statistics and Calculations

Heart rate was averaged over 15-s intervals. To calculate PPO (W) the values of the time of the test end were taken into consideration. The work done—Wtot (kJ)—was calculated on the basis of time and power output. This paper also presents heart rate values at loads of: 50 W, 100 W, 150 W, 200 W, and 250 W. HRR60 was calculated as the absolute difference between the HR measured at the time of termination of exercise and the HR in the 60 s of rest.

Statistical analysis was performed using Statistica 13.3 software (StatSoft Inc., Tulsa, OK, USA). Data were presented as means ($\bar{x}$) and standard deviations (± SD). The distribution of the data set was screened for normality using the Shapiro–Wilk test. The Student’s *t*-test was used in the evaluation of the differences between test protocols. For selected parameters, Pearson’s linear correlation coefficient was calculated. The level of α < 0.05 was considered statistically significant. Effect size (ES), that is Cohen’s d, was calculated in order to explore practical effect, using the following criteria: 0.1—trivial, 0.2—small, 0.5—medium, and 0.8—large.

## 3. Results

The test 0.278 W·s^−1^ was 8.5 min (*p* < 0.001; t = 28.31; ES = 4.14) longer than the second test. Peak power was significantly higher by 51.69 W (*p* < 0.001; t = 13.10; ES = 1.13) in the 0.556 W·s^−1^ test. The work done (Wtot) was significantly higher in the 0.278 W·s^−1^ test by 71.93 kJ (*p* < 0.001; t = 12.55; ES = 1.57). Maximal heart rate was significantly higher at 0.278 W·s^−1^ by 3.30 bpm (*p* < 0.01; t = 3.72; ES = 0.48) (Table 2). There were no differences in resting and peak blood lactate between the tests.

**Figure 2:**
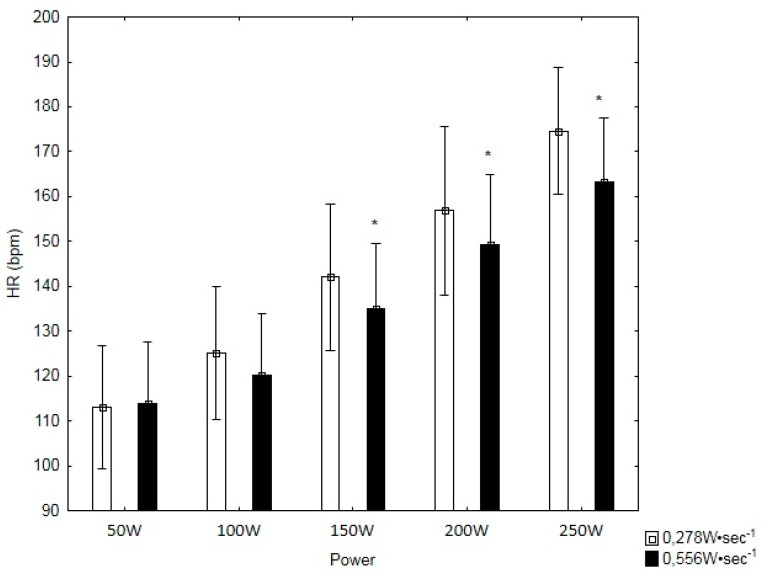
The submaximal heart rate (HR) values in power 50–250 W. *—statistically significant difference between tests (*p* < 0.05).

HR was significantly lower in 0.556 W·s^−1^ test for the intensity of 150 W by 7.05 bmp (*p* < 0.01; t = 3.09), 200 W by 7.70 bpm (*p* < 0.01; t = 3.12) and 250 W by 11.40 bpm (*p* < 0.001; t = 6.54) (Figure 2). There were no statistically significant differences in HRR60 between the tests.

%HRmax was significantly higher in 0.278 W·s^−1^ by 2.31% (*p* < 0.01, t = 3.09), by 2.60% (*p* < 0.01, t = 3.12) and by 4.36 (*p* < 0.001, t = 6.54) for intensity 150 W, 200 W, and 250 W, respectively (Table 3).

There was a statistically significant correlation between PPO (expressed as power per kilogram of body mass) and La^−^ for both tests: in the 0.278 W·s^−1^ test r = 0.52 (*p* < 0.05), while in the 0.556 W·s^−1^ test r = 0.67 (*p* < 0.001).

## 4. Discussion

This study succeeded in obtaining statistically significant higher peak power and lower work done in the test in which the power increased by 0.556 W·s^−1^. Moreover, there were no differences in the post-exercise lactate concentration in blood and rest values of heart rate. Times of the incremental test depended on the increase of intensity (slope of the load increase curve), with a greater slope shortening the time of work.

Peak power output (PPO) attained in endurance training strongly correlates with long-distance performance [20]. The results obtained in this research support our hypothesis and are consistent with those presented by Jamnick et al. [21]. They prove that the shorter time of incremental testing results in higher peak power. This may cause discrepancies in the determination of training intensity, e.g., HIIT interval training [22]. PPO values being higher by 15% in the 0.556 W·s^−1^ test may result from more efficient use of working muscles through a greater involvement of oxygen-dependent Type I fibers and a reduction in the involvement of Type II fibers in the initial phase of the test [23]. As a consequence, fatigue of Type II fibers does not occur prematurely and results in attaining higher PPO despite similar La^−^ concentration. On the one hand, no difference in La^−^ concentration may indicate a lack of greater involvement of aerobic metabolism. Conversely, the stronger correlation between La^−^ and PPO (r = 0.67) (expressed in kilograms per body mass) in the 0.556 W·s^−1^ test proves better efficiency of type II fibers under these conditions. It is consistent with the suggestion of Roffey et al. [24] regarding the lack of differences in the La^−^ concentration between tests. Different results, however, were presented by Machado et al. [8], who observed a significantly higher lactate concentration in the 11-min test as opposed to the 26-min test. But, the moment (in 5th min) at which blood lactate reached peak concentration after the incremental test, during passive recovery, was independent of the stage duration of the protocol. In our studies, the total work in the 0.278 W test was 66% of the total work obtained in the second test. This affects the physiological cost of work measured by the heart rate of work performed at sub-maximal power (50–250 W). The HR values were significantly lower in the 0.556 W·s^−1^ test at the intensity range of 150–250 W. Physiological responses to the same intensity of work during long-term efforts indicate higher physiological cost in comparison to short-term testing [24]. Yoon et al. [13] have suggested dehydration to be the main reason of increasing fatigue in longer tests.

Like peak power, heart rate (HR) expressed as a percentage of reserve of HRmax is often used to indicate aerobic training zones [24,25]. Therefore, it is known that differences in HRmax (as well as differences in physiological cost expressed in HR or %HRmax at work 50–250 W) lead to different intensity ranges in training (overestimation or underestimation). The results of our research proved that HRmax was higher in the longer test with a slower increase in intensity. This is in line with studies by Roffey et al. [24] and Bishop et al. [26], who have observed higher HRmax values for the longer incremental test. HRmax and La^−^ concentration met the criterion of attaining maximal exertion [2]. Moreover, if the difference between HRmax in the increase phase and the verification phase is ≤4 beats per minute, this is considered sufficient proof of maximal exertion [27]. Both tests carried out in this study met the mentioned criteria. Higher HR can be explained by the so-called cardiac drift caused by dehydration, which occurs during work lasting more than 10–15 min [28]. HR drifting upward may be connected with such factors as catecholamine and hyperthemia. This suggests that longer exercise increases metabolic cost and body temperature [29]. Boudet et al. [29] have claimed that long-lasting exercise stimulates the sympathetic nervous system, which causes additional rise in catecholamine concentration. It is possible that the use of an intermediate protocol with power set to 0.417 W·s^−1^ (75 W·3 min^−1^) and a work time of approx. 14–16 min would help to reach a compromise between the peak power and HR drift to obtain HRmax. Such an approach was represented by Machado et al. [8], who tested an incremental protocol with a 2-min test as an alternative to test durations of 1 and 3 min.

Post-exercise heart rate recovery has been studied by many researchers who evaluated the impact of many factors on the rate of HR decrease, such as: training status, fatigue, type of exercise, and age [30,31,32,33,34] However, HRR has never been evaluated after different types of incremental tests, which is commonly used to provoke maximal exertion [4]. We hypothesized that HRR after the 0.556 W·s^−1^ test, which was shorter and only 1/3 of the work was done, would be faster. However, the results obtained have proven that HRR does not depend on test protocol and time. The results are consistent with the suggestion of Suzic Lazic et al. [4], who have proven a minimal impact of the incremental test on the HRR. The possible explanation may be lack of differences in post-exercise La^−^ concentration. As stated by Buchheit et al. [35], effective HRR takes place due to lower glycolytic activity of muscles and higher oxidative capacity, accompanied by faster phosphocreatine resynthesis and faster regulation of acid–base balance. In another study by Buchheit et al. [36] it was found that, in addition to those factors mentioned above, the secretion of adrenaline was probably responsible for an increased background of the sympathetic activity of the autonomic nervous system. More detailed research is needed to explain the lack of differences in HRR after incremental testing. It should also include analysis of respiratory gases, acid–base balance, and hormone concentration.

Some limitations need to be addressed concerning the present study, despite the fact that the results were interesting. First is that the lack of measurements for maximal oxygen uptake, which is a universal indicator of the aerobic fitness in the incremental test [1,7]. This is necessary to check the tests used in this study based on the analysis of respiratory gases (ventilatory thresholds), and it should reference the maximal and sub-maximal values obtained in this test to the typical cycling efforts, e.g., individual time trials. This is necessary to determine the implicational value of measured variables. Future research should consider these suggestions, especially for athletes.

## 5. Conclusions

The most interesting result of this study was that the test with linearly increasing workload 0.556 W·s^−1^ allows to get higher values of PPO with a smaller amount of total work done compared to test 0.278 W·s^−1^. Interestingly, post-exercise blood lactate and heart rate recovery are independent of the intensity RAMP test protocol. In summary, we recommend use of the 0.556 W·s^−1^ RAMP protocol because it is of shorter duration compared with 0.278 W·s^−1^ and that way practically easier and of less effort for subjects.

## Figures and Tables

**Table 1:** Selected parameters of the tested males *n* = 20 ($\bar{x}$ ± SD).

Variables	Value
Age (years)	22.75 ± 2.88
Body height (cm)	181.90 ± 4.95
BM (kg)	79.73 ± 8.73
%FM (%)	17.65 ± 3.73
Physical activity (h per week)	9.13 ± 6.47

BM—body mass, %FM—percent fat mass.

**Table 2:** Variables attained in the incremental tests ($\bar{x}$ ± SD).

Variables	0.278 W·s^−1^	0.556 W·s^−1^
Time (min:s)	20:29 ± 2:36	11:47 ± 1:26 *
PPO (W)	341.31 ± 43.33	393.00 ± 47.79 *
PPO (W·kg^−1^)	4.33 ± 0.72	5.01 ± 0.81 *
Wtot (kJ)	212.89 ± 54.88	140.96 ± 34.62 *
HRrest (bpm)	87.62 ± 11.56	90.16 ± 13.96
HRmax (bpm)	196.05 ± 6.94	192.75 ± 6.84 *
HRR60 (bpm)	27.10 ± 9.83	24.65 ± 6.88
La^−^ (mmol·L^−1^)	13.05 ± 2.30	13.01 ± 2.45

PPO—peak power output, Wtot—work done, HRrest—resting heart rate before the tests, HRmax—maximal heart rate, HRR60—heart rate recovery in the 60 s of rest, La^−^—peak blood lactate, *—statistically significant difference between tests (*p* < 0.05).

**Table 3:** Percentage of maximal heart rate in submaximal intensities (50–250 W) ($\bar{x}$ ± SD).

Power (W)	0.278 W·s^−1^	0.556 W·s^−1^
50 (%)	57.69 ± 6.76	59.05 ± 6.64
100 (%)	63.85 ± 7.10	62.38 ± 6.69
150 (%)	72.37 ± 7.39	70.06 ± 7.03 *
200 (%)	79.94 ± 8.58	77.34 ± 7.18 *
250 (%)	89.01 ± 5.73	84.65 ± 6.35 *

*—statistically significant difference between tests (*p* < 0.05).
